# Assessment of acute, 14-day, and 13-week repeated oral dose toxicity of *Tiglium* seed extract in rats

**DOI:** 10.1186/s12906-018-2315-5

**Published:** 2018-09-12

**Authors:** Jun-Won Yun, Euna Kwon, Yun-Soon Kim, Seung-Hyun Kim, Ji-Ran You, Hyoung-Chin Kim, Jin-Sung Park, Jeong-Hwan Che, Sang-Koo Lee, Ja-June Jang, Hyeon Hoe Kim, Byeong-Cheol Kang

**Affiliations:** 1 Department of Biotechnology, The Catholic University of Korea, 43 Jibongro, Bucheon, 14662 Republic of Korea; 2 Department of Experimental Animal Research, Biomedical Research Institute, Seoul National University Hospital, 101 Daehak-ro, Jongno-gu, Seoul, 03080 Republic of Korea; 3 Laboratory Animal Resource Center, Korea Research Institute of Bioscience and Biotechnology, 30 Yeongudanji-ro, Ochang-eup, Cheongwon-gu, Cheongju-si, Chungcheongbuk-do 28116 Republic of Korea; 4 Biomedical Center for Animal Resource and Development, Seoul National University College of Medicine, 101 Daehak-ro, Jongno-gu, Seoul, 03080 Republic of Korea; 5 Department of Pathology, Seoul National University College of Medicine, 101 Daehak-ro, Jongno-gu, Seoul, 03080 Republic of Korea; 6 Department of Urology, Seoul National University College of Medicine, 101 Daehak-ro, Jongno-gu, Seoul, 03080 Republic of Korea; 7 Graduate School of Translational Medicine, Seoul National University College of Medicine, 101 Daehak-ro, Jongno-gu, Seoul, 03080 Republic of Korea; 8 Designed Animal and Transplantation Research Institute, Institute of GreenBio Science Technology, Seoul National University, 1447 Pyeongchang-daero, Daehwa-myeon, Pyeongchang-gun, Gangwon-do 25354 Republic of Korea; 9 Graduate School of Translational Medicine, Seoul National University College of Medicine, 101 Daehak-ro, Jongno-gu, Seoul, 110-744 Republic of Korea

**Keywords:** *Tiglium* seed, Acute, Subchronic, Toxicity

## Abstract

**Background:**

Seed of mature *Croton tiglium Linne*, also known as *Tiglium seed* (TS), has been widely used as a natural product due to its several health beneficial properties including anti-tumor and antifungal activities. Despite its ethnomedicinal beneficial properties, toxicological information regarding TS extract, especially its long-term toxicity, is currently limited. Therefore, the objective of the present study was to evaluate acute and subchronic toxicity of TS extract in rats after oral administration following test guidelines of the Organization for Economic Cooperation and Development (OECD).

**Methods:**

Toxicological properties of TS extract were evaluated by toxicity assays to determine its single-dose acute toxicity (125, 250, 500, 1000, or 2000 mg/kg), 14-day repeated-dose toxicity (125, 250, 500, 1000, or 2000 mg/kg) and 13-week repeated-dose toxicity (31.25, 62.5, 125, 250, and 500 mg/kg) in Sprague-Dawley rats and F344 rats. Hematological, serum biochemical, and histopathological parameters were analyzed to determine its median lethal dose (LD_50_) and no-observed-adverse-effect-level (NOAEL).

**Results:**

Oral single dose up to 2000 mg/kg of TS extract resulted in no mortalities or abnormal clinical signs. In 13-week toxicity study, TS extract exhibited no dose-related changes (mortality, body weight, food/water consumption, hematology, clinical biochemistry, organ weight, or histopathology) at dose up to 500 mg/kg, the highest dosage level suggested based on 14-day repeat-dose oral toxicity study.

**Conclusion:**

Acute oral LD_50_ of TS extract in rats was estimated to be greater than 2000 mg/kg. NOAEL of TS extract administered orally was determined to be 500 mg/kg/day in both male and female rats. Results from these acute and subchronic toxicity assessments of TS extract under Good Laboratory Practice regulations indicate that TS extract appears to be safe for human consumption.

**Electronic supplementary material:**

The online version of this article (10.1186/s12906-018-2315-5) contains supplementary material, which is available to authorized users.

## Background

*Croton*, a large genus belonging to family *Euphorbiaceae*, is widespread in tropical regions of Southeast Asia and China. Many previous studies have reported beneficial pharmacological activities of *Croton* species for treating diabetes, gastritis, and digestive disorders [1–5]. Sandoval et al. [6] have demonstrated that *Croton tiglium* extract possesses growth inhibitory or antiproliferative effects on cancer. Ethanolic extract of *Croton tiglium* is also known to exhibit antifungal activities for treating dermatophytes caused by *Trichophyton mentagrophytes, T. rubrum, and Epidermophyton floccosum* [7]. *Croton tiglium Linne* has been recorded as a traditional Chinese medicine for gastrointestinal disorders, rheumatism, headache, peptic ulcer, and visceral pain in the second century B.C. [8–11]. *Tiglium* seed (TS), a seed of mature *Croton tiglium Linne*, is known to contain croton oil extensively used as laxative or purgative [12, 13].

Plant-derived natural products are attracting significant attention around the world due to the perception that they are safe ‘natural’ drugs for promoting health without showing side effects. In addition, it is easy to obtain them from many grocery stores without having a prescription [14–20]. However, it has been demonstrated that many traditional beneficial herbs have genotoxic or systemic (liver and kidney) toxicity [21–25]. Thus, it is necessary to conduct comprehensive safety analysis under strict guidelines for herbal medicine available on the market. Although many people believe that oriental herbs are safe, data on systemic oral toxicity of TS extract as a natural therapeutic product with a variety of beneficial properties are lacking. Therefore, the objective of this study was to determine the median lethal dose (LD_50_) and no-observed-adverse-effect-level (NOAEL) of TS extract after oral administration in Sprague-Dawley (SD) rats and F344 rats by conducting single-dose acute toxicity and 13-week repeated-dose toxicity studies following Good Laboratory Practice (GLP) regulations [26] and Organization for Economic Cooperation and Development (OECD) test guideline [27].

## Methods

### Test substance and animals

TS collected from China were purchased at a Kyung-dong traditional herbal market (Seoul, Korea) and authenticated by Professor Chang Soo Yook of Kyung Hee University, an expert in the field of herbal medicine. Voucher specimen (SNUH-GLP 04014) has been deposited at the Department of Experimental Animal Research, Biomedical Research Institute, Seoul National University Hospital in Korea. Stepwise extraction of TS was performed using solvents of increasing polarity (hexane and ethyl acetate) [28]. Briefly, TS was ground into powder after peeling off testa. After removing oil from TS, hexane was added to TS and kept at room temperature for 24 h. They were then filtered through filter paper and dried in clean and dark area at room temperature. After adding ethyl acetate, extracts were stored for 24 h, filtered, and dried. Distilled water (DW) was added to the dried extract and stirred at 4 °C for 72 h. They were filtered with membrane paper (0.24 μm, Eyela Tokyo Rikakikai Co., Ltd., Tokyo, Japan) and concentrated by evaporation to half volume. Finally, the TS extract was frozen dried for 24 h and dissolved in 1% methylcellulose (MC) before oral administration.

Specific pathogen-free Sprague-Dawley (SD) rats and F344 rats were obtained from Orient Bio (Seongnam, Korea) and SLC (Hamamatsu, Japan), respectively. The animals were housed under controlled conditions (temperature, 22 ± 2 °C; humidity, 40–60%) in the experimental animal facility at Seoul National University Hospital accredited by AAALAC International (#001169) in accordance with Guide for the Care and Use of Laboratory Animals, 8th edition [29]. These animals were allowed free access to their diet (LabDiet 5002 Certified Rodent Diet, PMI Nutrition International, St. Louis, MO, USA) and tap water with a 12 h light:dark cycle. The rats were adapted to this environment for 1 week prior to study initiation. The animal studies were approved by Institutional Animal Care and Use Committee of the Biomedical Research Institute at Seoul National University Hospital and conducted under the guideline published by OECD [27] as well as GLP regulations for Nonclinical Laboratory Studies of Korea Food Drug Administration [26].

### Experimental design for single oral dose toxicity study

For single oral dose toxicity study, healthy male and female SD rats (7-week old) were randomly assigned to six groups (3/sex/group). Vehicle (1% MC) or graded doses of TS extract (125, 250, 500, 1000, and 2000 mg/kg of body weight) were administered to rats by oral gavage once at dose of 10 ml/kg of body weight. The rats were observed for mortality and clinical signs every hour for 6 h after dosing during the first 24 h and then once daily for a total of 14 days. Body weights were recorded on days 0, 1, 7, and 14 after the treatment. At study termination, all rats were euthanized by isoflurane (2% to 5%) inhalation and their organs were then collected for macroscopic necropsy examination.

### Experimental design for repeated oral dose toxicity study

For 14-day repeat-dose toxicity study, healthy male and female F344 rats (7-week old) were randomly assigned to six groups (5/sex/group). Vehicle (1% MC) or graded doses of TS extract (125, 250, 500, 1000, and 2000 mg/kg of body weight) were administered to rats by oral gavage once daily for 14 days at dose of 10 ml/kg of body weight. The rats were observed daily for mortality and clinical signs for 14 days. Body weights were recorded on days 0, 7, and 14 after the treatment. At study termination, all rats were euthanized by isoflurane (2% to 5%) inhalation and their organs were then collected for macroscopic necropsy examination. Organ weights were measured for the following: liver, kidney, testis, thymus, heart, and lung.

For 13-week repeat-dose toxicity study in accordance with OECD guideline 408 [27], healthy male and female F344 rats (7-week old) were randomly assigned to six groups (10/sex/group). Vehicle (1% MC) or graded doses of TS extract (31.25, 62.5, 125, 250, and 500 mg/kg of body weight) were administered to rats by oral gavage once daily for 13 weeks at dose of 10 ml/kg of body weight. The rats were observed daily for clinical signs including mortality, general appearance, and behavioral abnormality until terminal sacrifice. Body weights and food/water consumption were recorded weekly throughout the study. At study termination, all rats were euthanized by isoflurane (2% to 5%) inhalation for blood sample collection.

### Hematology and serum biochemistry

After blood samples were collected into EDTA tubes, the following hematological parameters were analyzed using a Vet abc™ Animal Blood Counter (Horiba ABX, Montpellier, France): white blood cell (WBC), red blood cell (RBC), hemoglobin (HGB), hematocrit (HCT), platelet (PLT), mean corpuscular volume (MCV), mean corpuscular hemoglobin (MCH), mean corpuscular hemoglobin concentration (MCHC), and differential WBC (neutrophils, eosinophils, basophils, lymphocytes, and monocytes). The following serum biochemistry parameters were also measured using serum immediately separated from whole blood samples collected into tubes without anticoagulant with an automatic chemistry analyzer (Hitachi 7070, Hitachi, Tokyo, Japan): blood urea nitrogen (BUN), total cholesterol (TC), total bilirubin (TB), total protein (TP), albumin, alkaline phosphatase (ALP), aspartate transaminase (AST), alanine transaminase (ALT), creatinine, and triglyceride (TG).

### Gross findings, organ weights, and histopathological assessments

At necropsy, the animals were sacrificed to analyze the gross and microscopic features of the internal organs. The liver, kidney, testis, thymus, heart, and lung were excised and weighed. Relative organ weight to terminal body weight was also calculated. Liver, kidney, adrenal gland, urinary bladder, spleen, pancreas, thymus, thyroid gland, parathyroid gland, trachea, esophagus, lung, heart, salivary gland, lymph node, stomach, duodenum, jejunum, ileum, colon, rectum, preputial gland, clitoral gland, skin, brain, pituitary gland, bone marrow, prostate, seminal vesicle, ovary, uterus, and vagina were examined macroscopically and fixed in 10% neutral buffered formalin for histopathological examination. Testis and eyes with Harderian glands were also fixed with Bouin’s solution and Davidson solution, respectively. The nasal cavity and femora were decalcified for up to 3 weeks. Formalin-fixed samples were embedded in paraffin, sectioned, and stained with hematoxylin-eosin for histological analysis using a light microscope (IX61, Olympus, Tokyo, Japan).

### Statistical analysis

Data of male and female rats were analyzed separately by one-way analysis of variance (ANOVA) followed by multiple comparison with Dunnett’s test using SPSS software version 19 (SPSS Inc., Chicago, IL, USA). Statistical significance level was set at *p* < 0.05.

## Results

### Single oral dose toxicity study

**Fig. 1:**
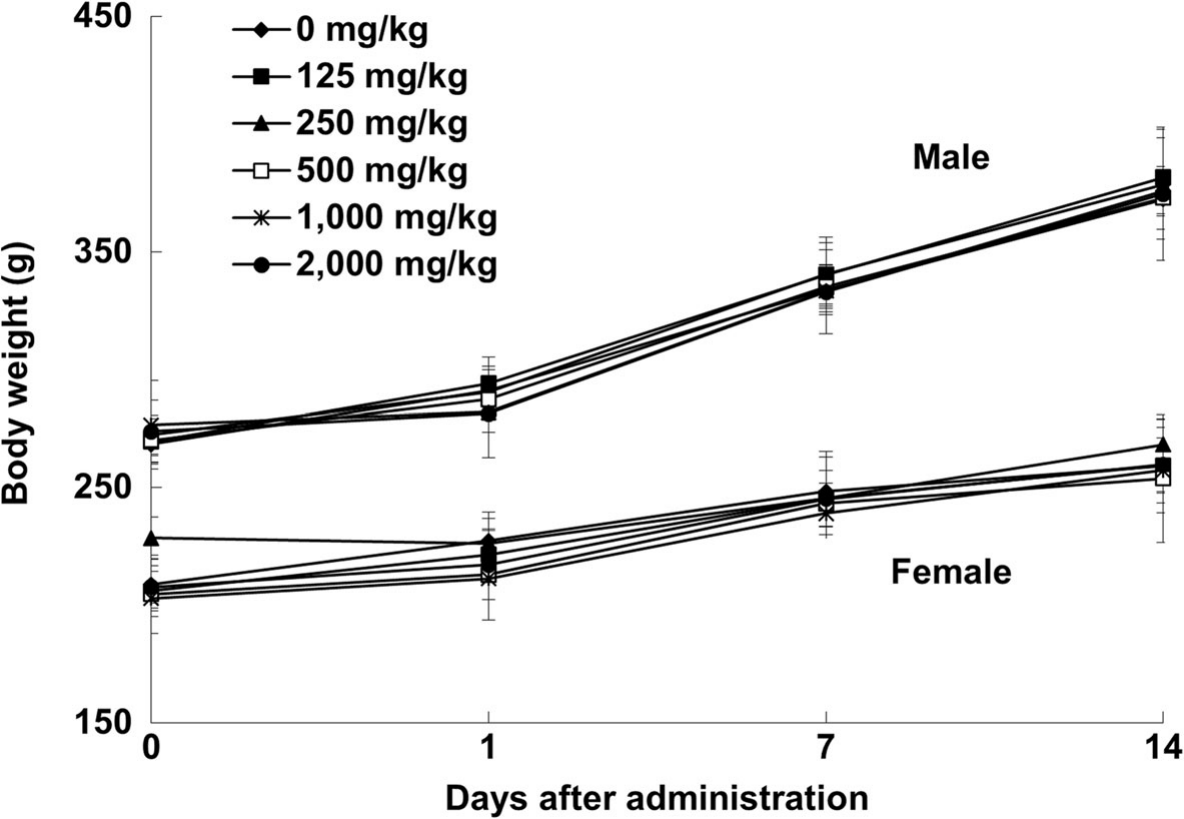
Body weight values of male and female SD rats orally administered with *Tiglium* seed extract in single dose toxicity study. Data are expressed as means ± SD (*n* = 3/sex/group)

In the 14-day observation of acute toxicity test using rats following a single oral administration of TS extract at 125, 250, 500, 1000, or 2000 mg/kg, no significant change in body weights attributable to the administration of TS extract was observed (Fig. 1). There were no significant changes in clinical signs, mortalities, or macroscopic necropsy examination of any organs at postmortem in either male or female rats following the administration of TS extract at any dose tested (data not shown).

### 14-day repeat-dose oral toxicity study

**Fig. 2:**
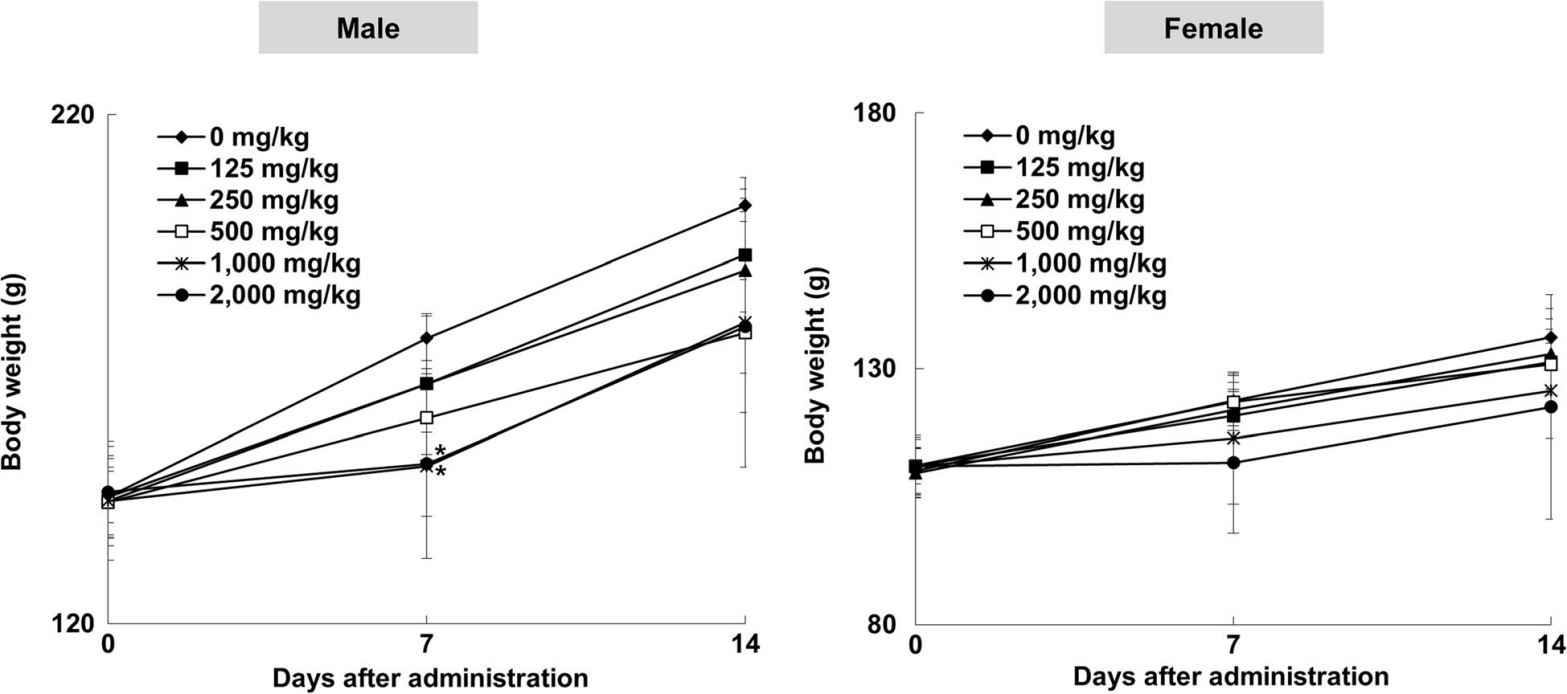
Body weight values of male and female F344 rats orally administered with *Tiglium* seed extract in 14-day repeat-dose toxicity study. Data are expressed as means ± SD (n = 3–5/sex/group). *Significantly different from Control group (*p* < 0.05)

Repeated administrations of TS extract at 125, 250, 500, 1000, and 2000 mg/kg by gavage for 14 days caused some side effects. As shown in Additional file 1, one male rat in the group treated with TS extract at 1000 mg/kg died at 13 days, showing clinical symptoms such as diarrhea and epistaxis. One male and two females in the group treated with TS extract at 2000 mg/kg also died at 7 days and 10 days, respectively, showing severe diarrhea and epistaxis. Although two male rats treated with 500 mg/kg of TS extract showed diarrhea at 6 days, they recovered within 12 h after symptom onset. Body weights of male rats in 1000 and 2000 mg/kg of TS extract treatment groups were significantly decreased compared to those in the negative control group at 7 days (Fig. 2). At the end of the 14-day observation period, macroscopic examination revealed no TS extract-related abnormality at necropsy in either sex of rats, although spontaneous lesions, such as congestion in lung and thymus, were sporadically observed without showing dose-dependency (Additional file 2). Absolute liver weights of male rats treated with TS extract at dose of 500 mg/kg (6.844 ± 0.820 g), 1000 mg/kg (6.983 ± 1.385 g), or 2000 mg/kg (7.486 ± 0.662 g) were significant decreased compared to those of negative control groups (8.716 ± 0.379 g) (Additional file 3). Similarly, female rats treated with TS extract at dose of 500 mg/kg (4.618 ± 0.268 g) or 1000 mg/kg (4.263 ± 0.205 g) showed significant decreases in absolute liver weights in comparison with those of negative control groups (5.560 ± 0.389 g). Relative liver weights of male rats treated with 500 mg/kg (3.860 ± 0.202%) or 1000 mg/kg (3.876 ± 0.200%) of TS extract were significantly lower than those of the negative control group (4.312 ± 0.177%). Relative weights of liver in female rats treated with 500 mg/kg (3.530 ± 0.150%), 1000 mg/kg (3.399 ± 0.140%), or 2000 mg/kg (4.446 ± 0.243%) of TS extract were also significantly changed compared with those in the negative control group (4.082 ± 0.178%). Of five TS extract concentrations tested, 500 mg/kg was selected as the highest dose of TS extract for 13-week repeat-dose oral toxicity study.

### 13-week repeat-dose oral toxicity study

#### General observation, body weight, feed intake, and water consumption

**Fig. 3:**
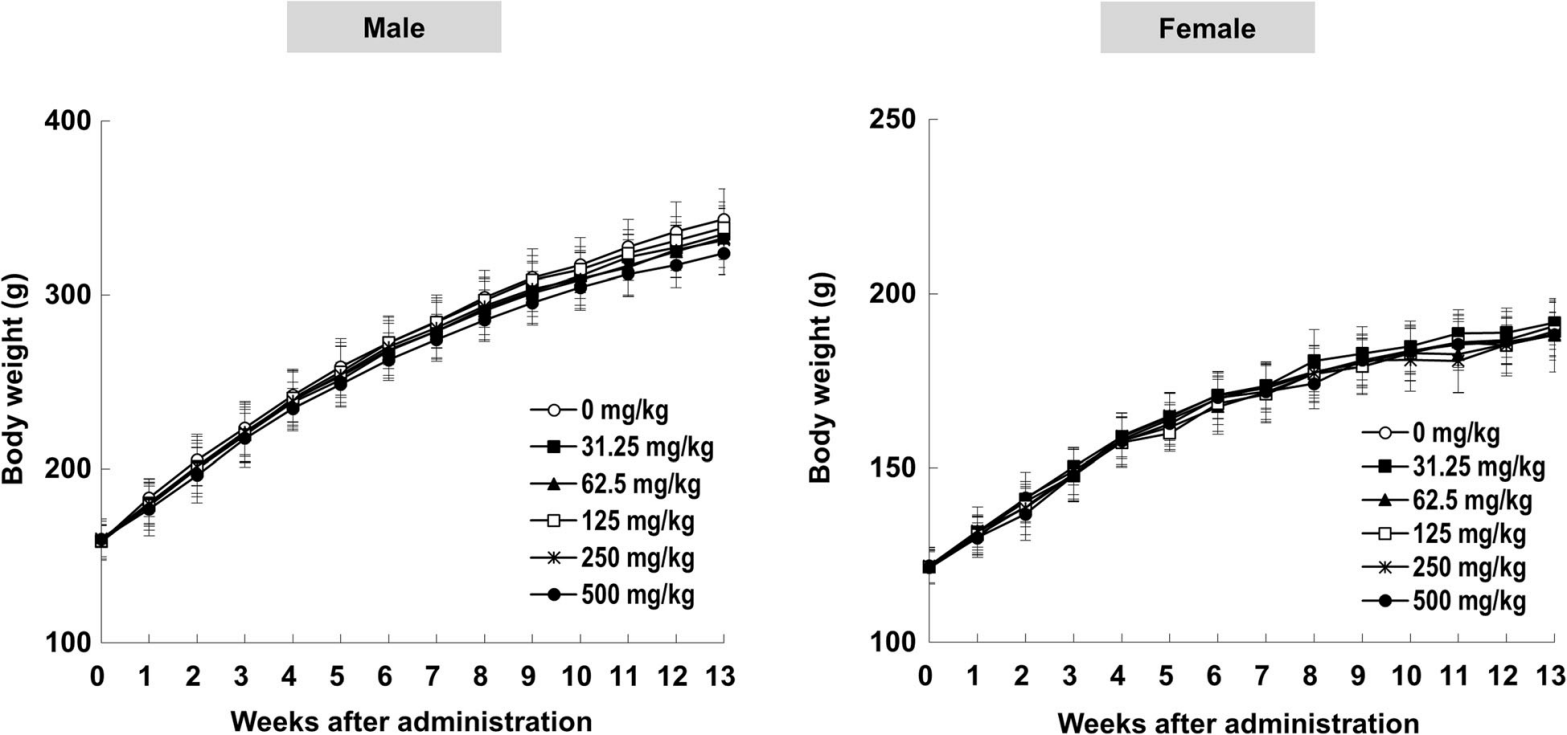
Body weight values of male and female F344 rats orally administered with *Tiglium* seed extract in 13-week repeat-dose toxicity study. Data are expressed as means ± SD (*n* = 9–10/sex/group)

During the study period for the subchronic toxicity trial, one female rat treated with TS extract at 125 mg/kg died at 39 days after administration. However, the death for this rat was not considered to be TS extract-related because gross and histological lesions were transient without showing dose-dependency. In clinical observation for exterior appearances and behavior, no adverse clinical signs attributable to TS extract were noted at any dose tested in males or females. Likewise, TS extract did not affect body weight gains at any dose tested throughout the study period (Fig. 3). Although statistically significant change in daily food intake of female rats receiving TS extract at 500 mg/kg was observed at 13 weeks (Additional file 4A), such change was considered as incidental since this was sporadic and minimal. There were no significant changes in water consumption for 13 weeks after TS extract administration among groups in either males or females (Additional file 4B).

#### Hematology and serum biochemistry

Results of hematological analysis are shown in Table 1. PLT levels in males treated with TS extract at dose of 250 mg/kg (614 ± 63.5) were significantly increased compared to those in the control group (528 ± 48.9). However, this finding was not regarded to be toxicologically relevant because this occurred infrequently without showing consistency between sexes.

**Table 1:** Hematological data of male and female F344 rats orally administered with *Tiglium* seed extract for 13 weeks

	Dose of *Tiglium* seed (mg/kg)					
	0^a^	31.25	62.5	125	250	500
Males						
WBC (10^3^/mm^3^)	6.1 ± 1.28	5.9 ± 0.90	6.5 ± 1.16	6.9 ± 1.37	6.4 ± 1.49	5.9 ± 1.60
RBC (10^6^/mm^3^)	8.46 ± 0.475	8.58 ± 0.282	8.52 ± 0.271	8.53 ± 0.351	8.69 ± 0.431	8.53 ± 0.279
HGB (g/dl)	14.1 ± 0.36	14.2 ± 0.26	14.2 ± 0.21	14.2 ± 0.37	14.4 ± 0.49	14.3 ± 0.40
HCT (%)	39.0 ± 2.10	39.4 ± 1.31	39.2 ± 1.37	39.6 ± 1.58	40.2 ± 2.10	39.6 ± 1.45
PLT (10^3^/mm^3^)	528 ± 48.9	565 ± 45.5	568 ± 37.2	556 ± 69.5	614 ± 63.5*	595 ± 36.4
MCV (fl)	46 ± 0.7	46 ± 0.5	46 ± 0.4	46 ± 0.5	46 ± 0.7	46 ± 0.5
MCH (pg)	16.7 ± 0.57	16.5 ± 0.42	16.7 ± 0.43	16.7 ± 0.42	16.6 ± 0.45	16.7 ± 0.43
MCHC (g/dl)	36.3 ± 1.20	36.0 ± 0.87	36.2 ± 0.96	35.9 ± 0.77	35.9 ± 0.88	36.1 ± 1.04
Reticulocytes (/100 RBC)	1.3 ± 0.54	1.1 ± 0.48	1.3 ± 0.31	1.3 ± 0.43	1.2 ± 0.37	1.1 ± 0.52
Neutrophils (%)	22 ± 7.0	19 ± 6.4	20 ± 5.0	20 ± 7.9	21 ± 7.4	23 ± 5.7
Eosinophils (%)	0 ± 0.4	1 ± 1.0	1 ± 0.6	0 ± 0.7	1 ± 0.7	1 ± 1.1
Basophils (%)	0 ± 0.0	0 ± 0.0	0 ± 0.0	0 ± 0.0	0 ± 0.0	0 ± 0.0
Lymphocytes (%)	77 ± 7.2	80 ± 6.9	79 ± 4.8	79 ± 8.0	78 ± 7.8	76 ± 5.9
Monocytes (%)	1 ± 1.1	1 ± 0.8	1 ± 1.0	1 ± 1.0	1 ± 0.7	1 ± 0.7
Females						
WBC (10^3^/mm^3^)	5.3 ± 0.92	4.9 ± 0.63	5.1 ± 0.69	5.1 ± 0.82	5.0 ± 1.23	4.3 ± 0.77
RBC (10^6^/mm^3^)	7.59 ± 0.380	7.55 ± 0.369	7.49 ± 0.346	7.67 ± 0.326	7.68 ± 0.309	7.48 ± 0.293
HGB (g/dl)	13.9 ± 0.40	14.0 ± 0.37	13.8 ± 0.27	14.1 ± 0.40	14.2 ± 0.33	13.8 ± 0.24
HCT (%)	37.3 ± 1.55	37.4 ± 1.64	37.0 ± 1.44	37.9 ± 1.50	38.1 ± 1.33	37.1 ± 1.06
PLT (10^3^/mm^3^)	625 ± 47.7	623 ± 27.2	620 ± 51.8	643 ± 60.8	649 ± 47.3	638 ± 81.4
MCV (fl)	49 ± 0.5	49 ± 0.7	50 ± 0.5	49 ± 0.4	50 ± 0.7	50 ± 0.7
MCH (pg)	18.3 ± 0.57	18.5 ± 0.53	18.4 ± 0.53	18.4 ± 0.50	18.5 ± 0.49	18.5 ± 0.65
MCHC (g/dl)	37.2 ± 0.75	37.4 ± 0.86	37.2 ± 0.76	37.2 ± 0.84	37.2 ± 0.77	37.3 ± 0.91
Reticulocytes (/100 RBC)	1.0 ± 0.25	1.4 ± 0.39	1.3 ± 0.41	1.2 ± 0.27	1.1 ± 0.43	1.3 ± 0.53
Neutrophils (%)	17 ± 6.0	13 ± 3.6	18 ± 4.2	16 ± 7.7	17 ± 5.6	20 ± 4.6
Eosinophils (%)	1 ± 1.0	1 ± 0.7	1 ± 0.6	1 ± 1.2	0 ± 0.3	1 ± 0.7
Basophils (%)	0 ± 0.0	0 ± 0.0	0 ± 0.0	0 ± 0.0	0 ± 0.0	0 ± 0.0
Lymphocytes (%)	81 ± 5.9	86 ± 4.2	81 ± 3.9	82 ± 7.1	82 ± 6.4	79 ± 4.8
Monocytes (%)	1 ± 1.1	1 ± 0.5	1 ± 0.7	1 ± 1.4	1 ± 0.9	1 ± 1.2

Data expressed as means ± SD

*Significantly different from Control group (*p* < 0.05)

^a^Control group

Serum biochemical analysis revealed that TC levels in females following TS extract treatment at 125 mg/kg (85 ± 8.9 mg/dL), 250 mg/kg (84 ± 7.2 mg/dL), or 500 mg/kg (81 ± 7.1 mg/dL) were significantly decreased than those of the negative control group (95 ± 7.4 mg/dL) (Table 2). Serum levels of TP were in rats treated with TS extract at 62.5 mg/kg (male: 7.1 ± 0.19 g/dL) or 125 mg/kg (male: 7.2 ± 0.25 g/dL; female: 6.2 ± 0.29 g/dL) were significantly changed compared to their respective negative control groups (male: 6.8 ± 0.18 g/dL; female: 6.5 ± 0.32 g/dL). Albumin levels were significantly increased in the group treated with 62.5 mg/kg of TS extract (3.2 ± 0.10 g/dL) in males but decreased in the group treated with 125 mg/kg of TS extract (2.8 ± 0.12 g/dL) in females compared with those in their respective negative control groups (male: 3.0 ± 0.05 g/dL; female: 3.0 ± 0.15 g/dL). Serum AST levels in the group treated with 500 mg/kg of TS extract (125 ± 27.2 IU/L) were significantly lower than those of the negative control group (172 ± 51.0 IU/L) in males. Serum levels of ALT after subchronic administration of TS extract at 500 mg/kg in females (55 ± 7.0 IU/L) were significantly decreased in comparison with those in the negative control group (71 ± 17.3 IU/L). Levels of TG in groups treated with TS extract at 125 mg/kg (59 ± 15.6 mg/dL) or 250 mg/kg (60 ± 10.4 mg/dL) were also significantly lower than those of the negative control group (81 ± 28.4 mg/dL) in females.

**Table 2:** Serum biochemistry data of male and female F344 rats orally administered with *Tiglium* seed extract for 13 weeks

	Dose of *Tiglium* seed (mg/kg)					
	0^a^	31.25	62.5	125	250	500
Males						
BUN (mg/dL)	22 ± 3.0	23 ± 3.7	24 ± 4.6	23 ± 3.8	22 ± 4.2	22 ± 4.0
TC (mg/dL)	71 ± 7.2	74 ± 5.7	73 ± 7.3	77 ± 5.8	69 ± 6.2	69 ± 6.8
TP (g/dL)	6.8 ± 0.18	7.0 ± 0.19	7.1 ± 0.19*	7.2 ± 0.25*	6.8 ± 0.20	6.8 ± 0.13
Albumin (g/dL)	3.0 ± 0.05	3.1 ± 0.09	3.2 ± 0.10*	3.2 ± 0.16	3.1 ± 0.10	3.1 ± 0.08
TB (mg/dL)	0.1 ± 0.00	0.1 ± 0.00	0.1 ± 0.00	0.1 ± 0.00	0.1 ± 0.00	0.1 ± 0.00
ALP (IU/L)	207 ± 22.8	219 ± 43.4	210 ± 26.1	201 ± 33.2	188 ± 22.3	189 ± 27.3
AST (IU/L)	172 ± 51.0	150 ± 29.5	161 ± 32.6	161 ± 31.8	132 ± 34.1	125 ± 27.2*
ALT (IU/L)	83 ± 22.4	70 ± 10.5	75 ± 12.2	81 ± 11.5	74 ± 11.8	67 ± 7.8
Creatinine (mg/dL)	0.6 ± 0.05	0.5 ± 0.05	0.6 ± 0.07	0.6 ± 0.07	0.5 ± 0.05	0.6 ± 0.05
TG (mg/dL)	194 ± 39.2	210 ± 35.8	178 ± 38.3	221 ± 82.7	180 ± 55.4	151 ± 40.7
Females						
BUN (mg/dL)	22 ± 5.5	21 ± 4.7	20 ± 3.9	19 ± 3.4	20 ± 4.4	18 ± 3.0
TC (mg/dL)	95 ± 7.4	93 ± 10.6	93 ± 8.5	85 ± 8.9*	84 ± 7.2*	81 ± 7.1*
TP (g/dL)	6.5 ± 0.32	6.6 ± 0.22	6.6 ± 0.15	6.2 ± 0.29*	6.3 ± 0.20	6.4 ± 0.21
Albumin (g/dL)	3.0 ± 0.15	3.0 ± 0.11	3.0 ± 0.08	2.8 ± 0.12*	2.9 ± 0.09	2.9 ± 0.12
TB (mg/dL)	0.1 ± 0.00	0.1 ± 0.00	0.1 ± 0.00	0.1 ± 0.00	0.1 ± 0.00	0.1 ± 0.00
ALP (IU/L)	176 ± 21.1	187 ± 19.9	199 ± 39.0	184 ± 32.7	182 ± 30.5	166 ± 27.9
AST (IU/L)	148 ± 35.1	152 ± 30.9	150 ± 29.4	134 ± 26.9	143 ± 33.8	137 ± 32.2
ALT (IU/L)	71 ± 17.3	70 ± 13.8	66 ± 6.9	63 ± 10.0	63 ± 9.1	55 ± 7.0*
Creatinine (mg/dL)	0.6 ± 0.10	0.6 ± 0.07	0.6 ± 0.05	0.6 ± 0.07	0.7 ± 0.07	0.6 ± 0.04
TG (mg/dL)	81 ± 28.4	66 ± 11.5	78 ± 22.0	59 ± 15.6*	60 ± 10.4*	63 ± 16.7

Data expressed as means ± SD

*Significantly different from Control group (*p* < 0.05)

^a^Control group

#### Organ weights and histopathological changes

Results of absolute and relative organ weights are shown in Table 3. Relative weights of testis were significantly increased in males treated with TS extract at 500 mg/kg (0.471 ± 0.036%) compared to those of the negative control group (0.426 ± 0.023%). Absolute weights of thymus in male rats treated with TS extract at 250 mg/kg (0.198 ± 0.017 g) or 500 mg/kg (0.190 ± 0.024 g) were significantly lower than those in the negative control group (0.228 ± 0.011 g). Relative heart weights of male rats treated with TS extract at dose of 500 mg/kg (0.295 ± 0.009%) were significant higher than those of the negative control group (0.282 ± 0.010%).

**Table 3:** Organ weights of male and female F344 rats orally administered with *Tiglium* seed extract for 13 weeks

		Dose of *Tiglium* seed (mg/kg)					
		0^a^	31.25	62.5	125	250	500
Males							
Liver	(g)	10.564 ± 0.488	10.654 ± 0.516	10.171 ± 0.727	10.782 ± 0.883	10.378 ± 0.862	9.816 ± 0.560
	(%BW)	3.188 ± 0.109	3.303 ± 0.162	3.195 ± 0.144	3.331 ± 0.204	3.301 ± 0.226	3.216 ± 0.179
Kidney	(g)	0.991 ± 0.023	0.981 ± 0.044	0.957 ± 0.063	0.987 ± 0.066	0.965 ± 0.087	0.934 ± 0.049
	(%BW)	0.300 ± 0.015	0.304 ± 0.017	0.301 ± 0.016	0.305 ± 0.012	0.307 ± 0.015	0.306 ± 0.012
Testis	(g)	1.408 ± 0.050	1.420 ± 0.066	1.396 ± 0.066	1.439 ± 0.075	1.426 ± 0.060	1.439 ± 0.119
	(%BW)	0.426 ± 0.023	0.440 ± 0.015	0.439 ± 0.023	0.445 ± 0.024	0.454 ± 0.024	0.471 ± 0.036*
Thymus	(g)	0.228 ± 0.011	0.231 ± 0.025	0.209 ± 0.027	0.206 ± 0.013	0.198 ± 0.017*	0.190 ± 0.024*
	(%BW)	0.069 ± 0.005	0.071 ± 0.005	0.066 ± 0.010	0.064 ± 0.004	0.063 ± 0.003	0.062 ± 0.008
Heart	(g)	0.937 ± 0.058	0.915 ± 0.039	0.933 ± 0.063	0.922 ± 0.049	0.910 ± 0.051	0.902 ± 0.047
	(%BW)	0.282 ± 0.010	0.283 ± 0.008	0.293 ± 0.009	0.285 ± 0.010	0.289 ± 0.007	0.295 ± 0.009*
Lung	(g)	1.234 ± 0.050	1.171 ± 0.069	1.181 ± 0.063	1.216 ± 0.125	1.181 ± 0.100	1.197 ± 0.071
	(%BW)	0.373 ± 0.016	0.363 ± 0.019	0.371 ± 0.016	0.377 ± 0.051	0.376 ± 0.022	0.392 ± 0.026
Females							
Liver	(g)	5.176 ± 0.348	5.293 ± 0.150	5.257 ± 0.249	5.212 ± 0.189	5.126 ± 0.350	5.152 ± 0.204
	(%BW)	2.844 ± 0.193	2.872 ± 0.090	2.917 ± 0.192	2.887 ± 0.160	2.849 ± 0.168	2.885 ± 0.106
Kidney	(g)	0.578 ± 0.039	0.580 ± 0.027	0.586 ± 0.026	0.577 ± 0.030	0.581 ± 0.034	0.586 ± 0.022
	(%BW)	0.317 ± 0.019	0.315 ± 0.016	0.324 ± 0.009	0.319 ± 0.012	0.323 ± 0.016	0.328 ± 0.010
Thymus	(g)	0.191 ± 0.008	0.192 ± 0.014	0.189 ± 0.014	0.193 ± 0.024	0.184 ± 0.024	0.185 ± 0.016
	(%BW)	0.105 ± 0.004	0.104 ± 0.006	0.105 ± 0.007	0.107 ± 0.015	0.102 ± 0.012	0.103 ± 0.009
Heart	(g)	0.579 ± 0.026	0.578 ± 0.024	0.575 ± 0.029	0.567 ± 0.044	0.562 ± 0.030	0.574 ± 0.021
	(%BW)	0.318 ± 0.015	0.314 ± 0.008	0.318 ± 0.010	0.313 ± 0.014	0.312 ± 0.011	0.321 ± 0.008
Lung	(g)	0.923 ± 0.112	0.822 ± 0.061	0.851 ± 0.055	0.868 ± 0.081	0.854 ± 0.036	0.855 ± 0.051
	(%BW)	0.507 ± 0.066	0.446 ± 0.031	0.471 ± 0.027	0.481 ± 0.055	0.475 ± 0.019	0.479 ± 0.037

Data expressed as means ± SD

*Significantly different from Control group (*p* < 0.05)

^a^Control group

In gross visual observation, organs of rats in TS extract treatment groups at all dose levels tested showed macroscopic pathologies similar to those of control rats (Additional file 5). Histopathological evaluation did not reveal dose-related abnormal symptoms in sampled organs or tissues of TS extract groups, although spontaneous lesions, including basophilic tubule in kidney (six out of 10 male control rats) and focal inflammation in heart (nine out of 10 male control rats) encountered more frequently in male control rats, occurred in several tissues of rats in both control and TS extract groups (Table 4).

**Table 4:** Results of microscopic observation of F344 rats orally administered with *Tiglium* seed extract for 13 weeks

		Dose of *Tiglium* seed (mg/kg)								Dose of *Tiglium* seed (mg/kg)					
		Male			Female					Male			Female		
		0^a^	250	500	0	250	500			0	250	500	0	250	500
Liver	Normal	10/10	10/10	10/10	10/10	10/10	10/10	Duodenum	Normal	10/10	10/10	10/10	10/10	10/10	10/10
Kidney	Normal	4/10	5/10	8/10	9/10	8/10	9/10	Jejunum	Normal	10/10	10/10	10/10	10/10	10/10	10/10
	Tubular calcification	0/10	0/10	0/10	1/10	2/10	1/10	Ileum	Normal	10/10	10/10	10/10	10/10	10/10	10/10
	Basophilic tubule	6/10	5/10	2/10	0/10	0/10	0/10	Colon	Normal	10/10	10/10	10/10	10/10	10/10	10/10
Thyroid gland	Normal	10/10	10/10	10/10	10/10	10/10	10/10	Rectum	Normal	10/10	10/10	10/10	10/10	10/10	10/10
Urinary bladder	Normal	10/10	10/10	10/10	10/10	10/10	10/10	Preputial/Clitoral gland	Normal	10/10	10/10	10/10	10/10	10/10	10/10
Spleen	Normal	10/10	10/10	10/10	10/10	10/10	10/10	Skin/ Mammary gland	Normal	10/10	10/10	10/10	10/10	10/10	10/10
Pancreas	Normal	10/10	10/10	10/10	10/10	10/10	10/10	Eye	Normal	10/10	10/10	10/10	10/10	10/10	10/10
Thymus	Normal	10/10	10/10	10/10	10/10	10/10	10/10	Harderian gland	Normal	8/10	10/10	10/10	10/10	4/10	9/10
Thyroid gland	Normal	10/10	10/10	10/10	10/10	10/10	10/10		Inflammation	0/10	0/10	0/10	0/10	1/10	0/10
Parathyroid gland	Normal	10/10	10/10	10/10	10/10	10/10	10/10		Focal inflammation	2/10	0/10	0/10	0/10	5/10	1/10
Trachea	Normal	10/10	10/10	10/10	10/10	10/10	10/10	Brain	Normal	10/10	10/10	10/10	10/10	10/10	10/10
Esophagus	Normal	10/10	10/10	10/10	10/10	10/10	10/10	Pituitary gland	Normal	10/10	10/10	10/10	10/10	10/10	10/10
Lung	Normal	0/10	0/10	2/10	3/10	1/10	2/10	Femur/Bone marrow	Normal	10/10	10/10	10/10	10/10	10/10	10/10
	Focal inflammation	10/10	10/10	8/10	3/10	8/10	8/10	Nasal cavity	Normal	10/10	10/10	10/10	10/10	10/10	10/10
	Pneumonia	0/10	0/10	0/10	4/10	1/10	0/10	Testis	Normal	10/10	10/10	10/10	–	–	–
Heart	Normal	0/10	4/10	3/10	7/10	8/10	8/10	Epididymis	Normal	10/10	10/10	10/10	–	–	–
	Focal inflammation	9/10	6/10	7/10	3/10	2/10	2/10	Prostate	Normal	10/10	10/10	10/10	–	–	–
	Focal myocarditis	1/10	0/10	0/10	0/10	0/10	0/10	Seminal vesicle	Normal	10/10	10/10	10/10	–	–	–
Salivary gland	Normal	10/10	10/10	10/10	10/10	10/10	10/10	Ovary	Normal	–	–	–	10/10	10/10	10/10
Cervical lymph node	Normal	10/10	10/10	10/10	10/10	10/10	10/10	Uterus	Normal	–	–	–	10/10	10/10	10/10
Mesenteric lymph node	Normal	10/10	10/10	10/10	10/10	10/10	10/10	Vagina	Normal	–	–	–	10/10	10/10	10/10
Stomach	Normal	10/10	10/10	10/10	10/10	10/10	10/10								

^a^Control group

## Discussion

TS is an important traditional medicine for treating constipation, dyspepsia, and dysenteria [8–11]. Mature *Croton tiglium* contains several anti-cancer components, including croton alkaloid, flavonoids, and diterpenes [30–32]. Croton oil from seeds of *Croton tiglium* is known to exert a variety of remarkable biological activities, including purgative, analgesic, antimicrobial, and inflammatory properties [8, 11]. It has also been particularly used for treating skin diseases including ringworm [33, 34]. Croton oil can modulate gastrointestinal motility and affect intestinal inflammation related to immunological milieu in mice [35]. Hu et al. [36] have also reported the effect of croton oil on spontaneous contraction of smooth muscle in rabbit jejunum. It has been shown that a pyrazine derivative crotonine isolated from leaves of *Croton tiglium* possesses analgesic effects [37].

In a previous study, data of in vitro chromosome aberration assay and in vivo micronucleus assay have demonstrated that TS extract has no clastogenic potential, although Ames test results have shown mutagenicity (base-substitution, frameshift, or cross-linking and oxidizing mutagen) of TS extract [38]. Genotoxicity, especially mutagenic potential, could be one initial risk factor closely involved in long-term carcinogenic pathway [39, 40]. Besides its mutagenicity, TS extract has also caused gap junctional intercellular communication (GJIC) dysfunction known to be involved in tumor promotion stage of carcinogenesis [38, 41]. Croton oil, one of main components of TS, contains many types of phorbol derivatives including 12-O-tetradecanoyl-phorbol-13-acetate (TPA) [4, 42] which act as a co-carcinogen or a tumor promoter [43], supporting the relationship between mutagenicity and GJIC inhibition of TS extract. Nonetheless, a comprehensive non-clinical subchronic toxicity study of TS extract under OECD guidelines and GLP regulation should be conducted for its safe use in humans.

In acute toxicity tests, single oral administration of TS extract at doses ranging from 0 to 2000 mg/kg showed no dose-related changes in mortality, body weights, or clinical signs, indicating that acute oral LD_50_ of TS extract was higher than 2000 mg/kg for both male and female rats. In 14 days of repeated-dose toxicity studies (0, 125, 250, 500, 1000, and 2000 mg/kg/day) conducted as a preliminary study for a 13-week repeated-dose toxicity study, four cases of death occurred in groups treated with TS extract at 1000 mg/kg and 2000 mg/kg (one male in 1000 mg/kg group, one male and two females in 2000 mg/kg group). Significant changes in parameters of body weights and organ weights were also observed in groups treated with TS extract at dose of 1000 mg/kg or more. Considering above results of meaningful toxicity in 14-day repeat-dose oral toxicity study, 500 mg/kg was selected as the highest-dose group of TS extract for the 13-week repeated-dose toxicity study.

In the 13-week subchronic toxicity study, repeated administrations of TS extract at dose up to 500 mg/kg did not cause abnormal alterations in mortality or behavioral symptoms as early signs of toxicity. Rats treated with TS extract did not show any dose-associated difference in terms of body weight or food/water consumption either, indicating that TS extract did not retard animal growth or normal metabolism. Furthermore, results of our current analysis for hematological parameters associated with systemic toxic symptoms (i.e., HCT, HGB, PLT, WBC, and RBC) also indicated no adverse changes in either sex of rats after treatment with the highest dosage of TS extract.

Liver and kidney are major organs involved in the metabolism and elimination of drugs and other foreign compounds. They can be considered as major target organs that suffer systemic adverse reactions following oral administration of drugs [44, 45]. Serum concentrations of BUN and creatinine are the most widely used indicators in clinical biochemistry test for monitoring renal function [46, 47]. Along with unchanged levels of serum biochemical parameters (BUN and creatinine) and kidney weights, pathology data (gross findings and histopathological findings) supported our conclusion that no renal toxicity could be attributed to TS extract treatment. The liver plays an important role in the metabolism, elimination, and detoxification of drugs or other agents [48]. Serum levels of four enzymes (ALT, AST, ALP, and TG) are commonly used as clinical biochemistry markers associated with liver damage. Among these enzymes, serum levels of ALP known to be increased in response to biliary obstruction [49] did not show any significant changes between negative control group and TS extract groups in male or female rats. Significant changes in other liver function markers such as ALT, AST (hepatocellular damage marker) [50, 51], and TG (fatty liver marker) [52] were observed in TS extract treatment groups, including decreased AST in males of 500 mg/kg group, decreased ALT in females of 500 mg/kg group, and decreased TG in females of 125 mg/kg and 250 mg/kg groups. However, these changes were considered incidental because they were within acceptable ranges (AST in males, 103 ± 14.2 IU/L; ALT in female, 52 ± 11.7 IU/L; TG in females, 65 ± 34.3 mg/dL; SLC, http://www.jslc.co.jp/pdf/rat/005_F3442013.pdf). In addition, they were not sex- or dose-related. Combining organ weights with macroscopic/microscopic examination results, certain organ weights were significantly changed while no pathological (histopathological) changes in these organs were found, indicating that TS extract did not produce any toxic symptoms of internal major organs, including liver, kidney, lung, spleen, pancreas, or brain. Taken together, subchronic exposure of rats to TS extract exhibited no treatment-related changes at dose up to 500 mg/kg. This corresponds to about 81 mg/kg in human by using the conversion help according to dosing adjustment guidelines of the US Food and Drug Administration [53].

## Conclusions

Results of single and 13-week repeated oral dose toxicity studies clearly supported that administration of TS extract did not exert adverse effect in male or female rats for most toxicological factors. In particular, acute exposure to TS extract did not exert any significant toxic influence on rats at any dose tested, suggesting that oral LD_50_ of TS extract was greater than 2000 mg/kg for both sexes. Subchronic exposure of rats to TS extract did not induce treatment-related damage to target organs, indicating that subchronic NOAEL of TS extract was 500 mg/kg for both male and female rats. The current study is the first to provide important background on safety concerns of TS extract by analyzing the traditional acute toxicity and subchronic toxicity profile of TS extract comprehensively under OECD guidelines and GLP regulations for human safe consumption.

## Additional files

Additional file 1: Survival of male and female F344 rats orally administered with *Tiglium* seed extract for 14 days. (TIF 630 kb)

Additional file 2: Summary incidence of gross findings of F344 rats orally administered with *Tiglium* seed extract for 14 days. (DOCX 17 kb)

Additional file 3: Organ weights of male and female F344 rats orally administered with *Tiglium* seed extract for 14 days. (DOCX 30 kb)

Additional file 4: Effects of *Tiglium* seed extract on the daily food intake and water consumption after oral administration in male and female rats for 13 weeks. (TIF 806 kb)

Additional file 5: Summary incidence of gross findings of F344 rats orally administered with *Tiglium* seed extract for 13 weeks. (DOCX 18 kb)

## Abbreviations

ALP: Alkaline phosphatase
ALT: Alanine transaminase
AST: Aspartate transaminase
BUN: Blood urea nitrogen
GJIC: Gap junctional intercellular communication
GLP: Good Laboratory Practice
HCT: Hematocrit
HGB: Hemoglobin
LD_50_: Median lethal dose
MC: Methylcellulose
MCH: Mean corpuscular hemoglobin
MCHC: Mean corpuscular hemoglobin concentration
MCV: Mean corpuscular volume
NOAEL: No-observed-adverse-effect-level
OECD: Organization for Economic Co-operation and Development
PLT: Platelet
RBC: Red blood cell
SD: Sprague-Dawley
TB: Total bilirubin
TC: Total cholesterol
TG: Triglyceride
TP: Total protein
TS: *Tiglium* seed
WBC: White blood cell
