# Supplementary figures and images for: Assessment of acute, 14-day, and 13-week repeated oral dose toxicity of Tiglium seed extract in rats
Source: BMC Complement Altern Med. 2018 Sep 12;18:251. doi: 10.1186/s12906-018-2315-5 (PMC6134578; doi:10.1186/s12906-018-2315-5)

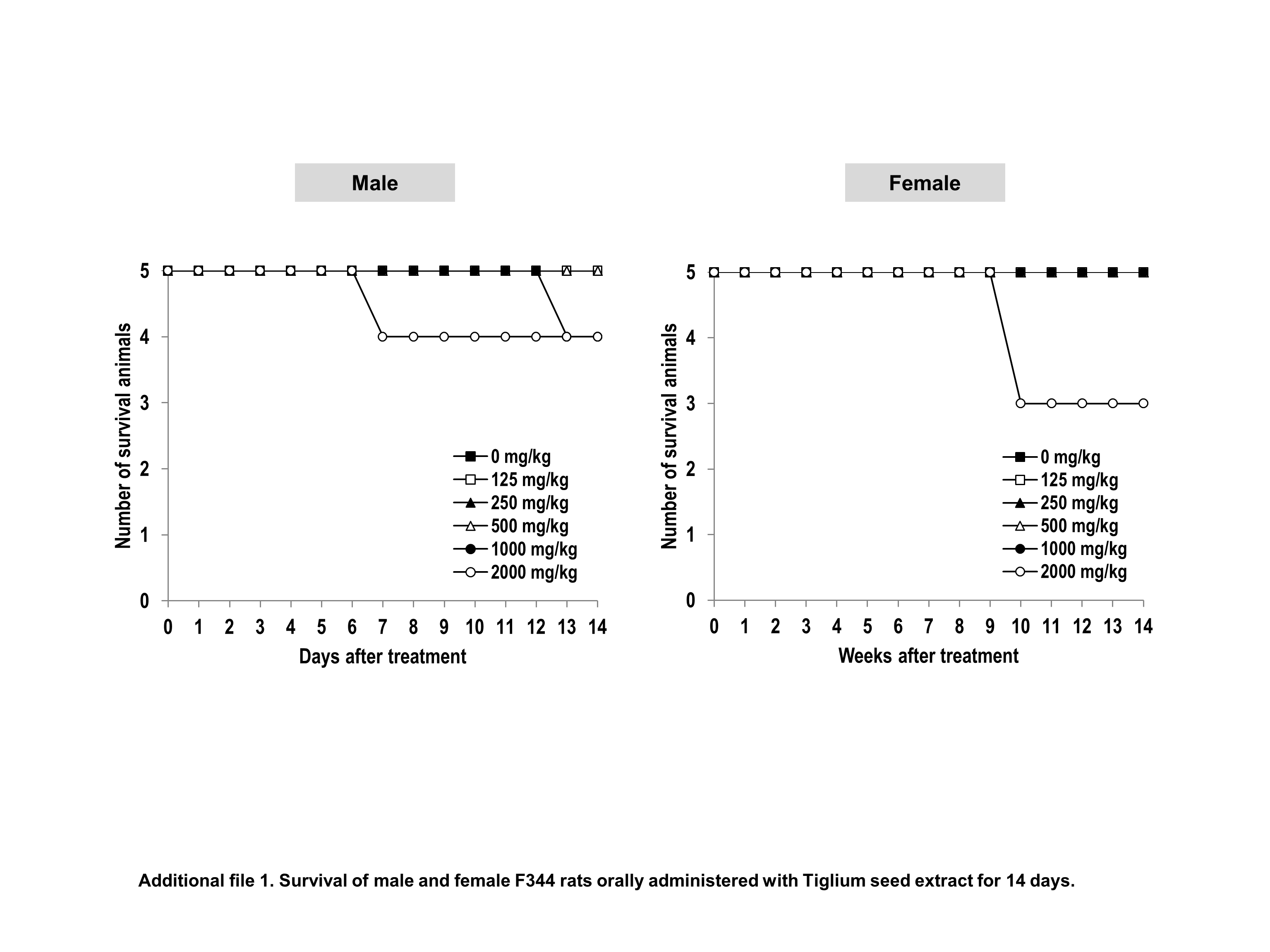

Supplement: Supplementary file 1 — Survival of male and female F344 rats orally administered with Tiglium seed extract for 14 days. (TIF 630 kb) [file 12906_2018_2315_MOESM1_ESM.tif]

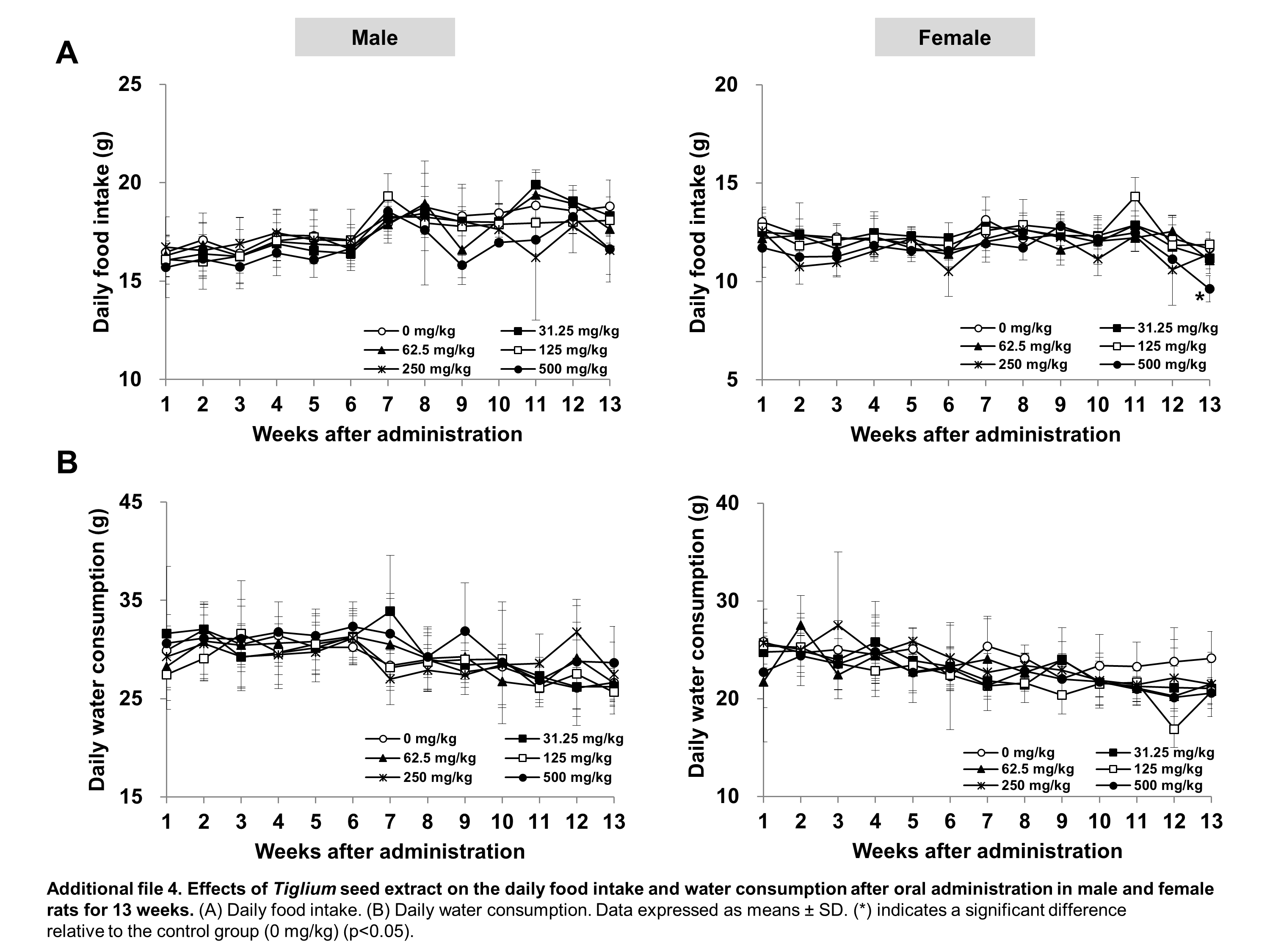

Supplement: Supplementary file 4 — Effects of Tiglium seed extract on the daily food intake and water consumption after oral administration in male and female rats for 13 weeks. (TIF 806 kb) [file 12906_2018_2315_MOESM4_ESM.tif]
