# Supplementary material for: Assessment of acute, 14-day, and 13-week repeated oral dose toxicity of Tiglium seed extract in rats
Source: BMC Complement Altern Med. 2018 Sep 12;18:251. doi: 10.1186/s12906-018-2315-5 (PMC6134578; doi:10.1186/s12906-018-2315-5)
Supplement: Supplementary file 2 — Summary incidence of gross findings of F344 rats orally administered with Tiglium seed extract for 14 days. (DOCX 17 kb) [file 12906_2018_2315_MOESM2_ESM.docx]

**Additional file 2. Summary incidence of gross findings of F344 rats orally administered with *Tiglium* seed extract for 14 days.**

|  | | **Dose of *Tiglium* seed (mg/kg)** | | | | | | | | | | | |
| --- | --- | --- | --- | --- | --- | --- | --- | --- | --- | --- | --- | --- | --- |
|  |  | **Male** | | | | | | **Female** | | | | | |
|  |  | **0^a^** | **125** | **250** | **500** | **1000** | **2000** | **0^a^** | **125** | **250** | **500** | **1000** | **2000** |
| Lung | Congestion | 0/5 | 1/5 | 0/5 | 1/5 | 0/4 | 0/4 | 1/5 | 0/5 | 0/5 | 0/5 | 0/5 | 0/3 |
|  | Foci | 1/5 | 0/5 | 0/5 | 1/5 | 0/4 | 0/4 | 0/5 | 1/5 | 0/5 | 1/5 | 0/5 | 0/3 |
| Thymus | Congestion | 1/5 | 1/5 | 0/5 | 0/5 | 1/4 | 1/4 | 0/5 | 0/5 | 1/5 | 0/5 | 0/5 | 1/3 |
|  | Foci | 0/5 | 0/5 | 1/5 | 0/5 | 0/4 | 0/4 | 0/5 | 0/5 | 0/5 | 0/5 | 0/5 | 0/3 |
|  | Atrophy | 0/5 | 0/5 | 0/5 | 1/5 | 1/4 | 0/4 | 0/5 | 0/5 | 0/5 | 0/5 | 1/5 | 0/3 |
| Liver | Nodule | 0/5 | 1/5 | 0/5 | 0/5 | 0/4 | 0/4 | 0/5 | 0/5 | 0/5 | 0/5 | 0/5 | 0/3 |
| Skin | Hemorrhage | 0/5 | 1/5 | 0/5 | 1/5 | 0/4 | 0/4 | 0/5 | 0/5 | 0/5 | 0/5 | 0/5 | 0/3 |
| Brain | Hemorrhage | 0/5 | 0/5 | 0/5 | 0/5 | 1/4 | 0/4 | 0/5 | 0/5 | 0/5 | 0/5 | 1/5 | 0/3 |
| Salivary gland (right) | Congestion | 0/5 | 0/5 | 0/5 | 0/5 | 1/4 | 0/4 | 0/5 | 0/5 | 0/5 | 0/5 | 0/5 | 0/3 |

^a^Control group.
