# Supplementary material for: Assessment of acute, 14-day, and 13-week repeated oral dose toxicity of Tiglium seed extract in rats
Source: BMC Complement Altern Med. 2018 Sep 12;18:251. doi: 10.1186/s12906-018-2315-5 (PMC6134578; doi:10.1186/s12906-018-2315-5)
Supplement: Supplementary file 3 — Organ weights of male and female F344 rats orally administered with Tiglium seed extract for 14 days. (DOCX 30 kb) [file 12906_2018_2315_MOESM3_ESM.docx]

**Additional file 3. Organ weights of male and female F344 rats orally administered with *Tiglium* seed extract for 14 days**

|  |  | **Dose of *Tiglium* seed (mg/kg)** | | | | | | | | |  |
| --- | --- | --- | --- | --- | --- | --- | --- | --- | --- | --- | --- |
|  |  | **0^a^** | **125** | **250** | **500** | | **1000** | | **2000** | |  |
| *Males* | |  |  |  |  | |  | |  | |  |
| Liver | (g) | 8.716 ± 0.379 | 7.960 ± 0.558 | 7.761 ± 0.647 | | 6.844 ± 0.820* | | 6.983 ± 1.385* | | 7.486 ± 0.662* | |
|  | (%BW) | 4.312 ± 0.177 | 4.137 ± 0.108 | 4.094 ± 0.152 | | 3.860 ± 0.202* | | 3.876 ± 0.200* | | 4.193 ± 0.210 | |
| Kidney | (g) | 0.796 ± 0.065 | 0.781 ± 0.055 | 0.771 ± 0.059 | | 0.721 ± 0.100 | | 0.701 ± 0.137 | | 0.670 ± 0.028 | |
|  | (%BW) | 0.394 ± 0.030 | 0.406 ± 0.016 | 0.407 ± 0.018 | | 0.406 ± 0.027 | | 0.390 ± 0.024 | | 0.376 ± 0.011 | |
| Testis | (g) | 1.091 ± 0.017 | 1.125 ± 0.029 | 1.093 ± 0.031 | | 1.053 ± 0.014 | | 1.040 ± 0.077 | | 1.085 ± 0.064 | |
|  | (%BW) | 0.540 ± 0.006 | 0.586 ± 0.032 | 0.578 ± 0.024 | | 0.598 ± 0.051 | | 0.588 ± 0.061 | | 0.609 ± 0.043 | |
| Thymus | (g) | 0.372 ± 0.011 | 0.336 ± 0.033 | 0.352 ± 0.037 | | 0.291 ± 0.094 | | 0.289 ± 0.117 | | 0.266 ± 0.099 | |
|  | (%BW) | 0.184 ± 0.007 | 0.174 ± 0.008 | 0.186 ± 0.020 | | 0.162 ± 0.045 | | 0.156 ± 0.051 | | 0.148 ± 0.052 | |
| Heart | (g) | 0.675 ± 0.015 | 0.662 ± 0.042 | 0.648 ± 0.060 | | 0.599 ± 0.042 | | 0.625 ± 0.093 | | 0.611 ± 0.048 | |
|  | (%BW) | 0.334 ± 0.007 | 0.344 ± 0.006 | 0.342 ± 0.015 | | 0.339 ± 0.011 | | 0.349 ± 0.011 | | 0.342 ± 0.010 | |
| Lung | (g) | 0.907 ± 0.054 | 0.904 ± 0.058 | 0.869 ± 0.050 | | 0.852 ± 0.063 | | 0.828 ± 0.040 | | 0.847 ± 0.080 | |
|  | (%BW) | 0.449 ± 0.027 | 0.471 ± 0.029 | 0.459 ± 0.008 | | 0.482 ± 0.025 | | 0.470 ± 0.063 | | 0.474 ± 0.031 | |
|  |  |  |  |  | |  | |  | |  | |
| *Females* | |  |  |  |  | |  | |  | |  |
| Liver | (g) | 5.560 ± 0.389 | 5.157 ± 0.243 | 5.247 ± 0.544 | | 4.618 ± 0.268* | | 4.263 ± 0.205* | | 5.421 ± 0.841 | |
|  | (%BW) | 4.082 ± 0.178 | 3.931 ± 0.133 | 3.943 ± 0.246 | | 3.530 ± 0.150* | | 3.399 ± 0.140* | | 4.446 ± 0.243* | |
| Kidney | (g) | 0.543 ± 0.025 | 0.527 ± 0.038 | 0.542 ± 0.056 | | 0.535 ± 0.041 | | 0.491 ± 0.028 | | 0.535 ± 0.045 | |
|  | (%BW) | 0.399 ± 0.007 | 0.402 ± 0.011 | 0.407 ± 0.022 | | 0.409 ± 0.025 | | 0.392 ± 0.026 | | 0.442 ± 0.048 | |
| Thymus | (g) | 0.315 ± 0.024 | 0.304 ± 0.025 | 0.297 ± 0.008 | | 0.317 ± 0.026 | | 0.256 ± 0.076 | | 0.231 ± 0.142 | |
|  | (%BW) | 0.232 ± 0.016 | 0.232 ± 0.020 | 0.224 ± 0.010 | | 0.243 ± 0.024 | | 0.202 ± 0.057 | | 0.178 ± 0.093 | |
| Heart | (g) | 0.503 ± 0.017 | 0.506 ± 0.018 | 0.493 ± 0.027 | | 0.483 ± 0.026 | | 0.488 ± 0.040 | | 0.460 ± 0.083 | |
|  | (%BW) | 0.370 ± 0.007 | 0.386 ± 0.011 | 0.371 ± 0.007 | | 0.369 ± 0.018 | | 0.388 ± 0.021 | | 0.376 ± 0.015 | |
| Lung | (g) | 0.748 ± 0.026 | 0.713 ± 0.026 | 0.712 ± 0.050 | | 0.727 ± 0.062 | | 0.700 ± 0.040 | | 0.717 ± 0.064 | |
|  | (%BW) | 0.551 ± 0.040 | 0.544 ± 0.027 | 0.536 ± 0.032 | | 0.556 ± 0.036 | | 0.558 ± 0.019 | | 0.594 ± 0.083 | |

^a^Control group.

Data expressed as means ± SD.

*Significantly different from Control group (*p*<0.05).
