# Supplementary material for: Assessment of acute, 14-day, and 13-week repeated oral dose toxicity of Tiglium seed extract in rats
Source: BMC Complement Altern Med. 2018 Sep 12;18:251. doi: 10.1186/s12906-018-2315-5 (PMC6134578; doi:10.1186/s12906-018-2315-5)
Supplement: Supplementary file 5 — Summary incidence of gross findings of F344 rats orally administered with Tiglium seed extract for 13 weeks. (DOCX 18 kb) [file 12906_2018_2315_MOESM5_ESM.docx]

**Additional file 5. Summary incidence of gross findings of F344 rats orally administered with *Tiglium* seed extract for 13 weeks.**

|  | | **Dose of *Tiglium* seed (mg/kg)** | | | | | | | | | | | |
| --- | --- | --- | --- | --- | --- | --- | --- | --- | --- | --- | --- | --- | --- |
|  |  | **Male** | | | | | | **Female** | | | | | |
|  |  | **0^a^** | **31.25** | **62.5** | **125** | **250** | **500** | **0^a^** | **31.25** | **62.5** | **125** | **250** | **500** |
| Liver | Nodule | 0/10 | 0/10 | 0/10 | 0/10 | 0/10 | 1/10 | 1/10 | 0/10 | 0/10 | 0/10 | 0/10 | 0/10 |
|  | Spot | 0/10 | 1/10 | 0/10 | 0/10 | 0/10 | 0/10 | 0/10 | 0/10 | 0/10 | 0/10 | 1/10 | 0/10 |
|  | Congestion | 0/10 | 0/10 | 0/10 | 0/10 | 0/10 | 0/10 | 0/10 | 0/10 | 0/10 | 1/10 | 0/10 | 0/10 |
|  | Hemorrhage | 0/10 | 0/10 | 0/10 | 0/10 | 1/10 | 0/10 | 0/10 | 0/10 | 0/10 | 0/10 | 0/10 | 0/10 |
| Lung | Spot | 1/10 | 1/10 | 1/10 | 2/10 | 1/10 | 0/10 | 0/10 | 0/10 | 0/10 | 0/10 | 1/10 | 0/10 |
|  | Congestion | 5/10 | 6/10 | 4/10 | 4/10 | 5/10 | 7/10 | 8/10 | 4/10 | 8/10 | 5/10 | 4/10 | 5/10 |
| Brain | Spot | 0/10 | 0/10 | 0/10 | 0/10 | 1/10 | 0/10 | 0/10 | 0/10 | 0/10 | 0/10 | 0/10 | 0/10 |
|  | Congestion | 0/10 | 0/10 | 0/10 | 1/10 | 0/10 | 0/10 | 0/10 | 0/10 | 0/10 | 0/10 | 0/10 | 0/10 |
|  | Hemorrhage | 2/10 | 0/10 | 0/10 | 0/10 | 2/10 | 1/10 | 0/10 | 0/10 | 0/10 | 1/10 | 0/10 | 0/10 |
| Glabella | Hemorrhage | 0/10 | 0/10 | 0/10 | 0/10 | 0/10 | 0/10 | 1/10 | 0/10 | 0/10 | 0/10 | 0/10 | 0/10 |
| Thymus | Spot | 0/10 | 0/10 | 0/10 | 0/10 | 0/10 | 1/10 | 0/10 | 0/10 | 0/10 | 3/10 | 2/10 | 1/10 |
| Cervical lymph node | Congestion | 0/10 | 0/10 | 0/10 | 0/10 | 0/10 | 0/10 | 0/10 | 0/10 | 0/10 | 1/10 | 0/10 | 0/10 |
| Uterus | Swelling | 0/10 | 0/10 | 0/10 | 0/10 | 0/10 | 0/10 | 2/10 | 0/10 | 0/10 | 2/10 | 0/10 | 1/10 |
| Small intestine | Congestion | 0/10 | 0/10 | 0/10 | 0/10 | 0/10 | 0/10 | 0/10 | 0/10 | 0/10 | 1/10 | 0/10 | 0/10 |
| Cecum | Congestion | 2/10 | 0/10 | 0/10 | 1/10 | 2/10 | 0/10 | 0/10 | 0/10 | 0/10 | 0/10 | 0/10 | 0/10 |
|  | Hemorrhage | 0/10 | 0/10 | 0/10 | 0/10 | 0/10 | 0/10 | 0/10 | 0/10 | 0/10 | 1/10 | 0/10 | 0/10 |
| Ileum | Congestion | 0/10 | 0/10 | 0/10 | 1/10 | 1/10 | 0/10 | 0/10 | 1/10 | 0/10 | 0/10 | 0/10 | 1/10 |
|  | Hemorrhage | 0/10 | 0/10 | 0/10 | 1/10 | 0/10 | 0/10 | 0/10 | 0/10 | 0/10 | 0/10 | 0/10 | 0/10 |

^a^Control group.
